# Intravitreal injection of mitochondrial DNA induces cell damage and retinal dysfunction in rats

**DOI:** 10.1186/s40659-022-00390-6

**Published:** 2022-06-03

**Authors:** Yue Guo, Dekang Gan, Fangyuan Hu, Yun Cheng, Jian Yu, Boya Lei, Qinmeng Shu, Ruiping Gu, Gezhi Xu

**Affiliations:** 1 Department of Ophthalmology, Eye and ENT Hospital of Fudan University, Shanghai, 200031 China; 2 Shanghai Key Laboratory of Visual Impairment and Restoration, Shanghai, 200031 China; 3 Key Laboratory of Myopia of State Health Ministry, Shanghai, 200031 China; 4 Eye Institute, Eye and ENT Hospital of Fudan University, Shanghai, 200031 China

**Keywords:** Mitochondrial DNA, cGAS–STING, Apoptosis, Retina

## Abstract

**Background:**

Retinal neurodegeneration is induced by a variety of environmental insults and stresses, but the exact mechanisms are unclear. In the present study, we explored the involvement of cytosolic mitochondrial DNA (mtDNA), resulting in the cGAS-STING dependent inflammatory response and apoptosis in retinal damage in vivo.

**Methods:**

Retinal injury was induced with white light or intravitreal injection of lipopolysaccharide (LPS). After light- or LPS-induced injury, the amount of cytosolic mtDNA in the retina was detected by PCR. The mtDNA was isolated and used to transfect retinas in vivo. WB and real-time PCR were used to evaluate the activation of cGAS-STING pathway and the levels of apoptosis-associated protein at different times after mtDNA injection. Retinal cell apoptosis rate was detected by TUNEL staining. Full-field electroretinography (ERG) was used to assess the retinal function.

**Results:**

Light injury and the intravitreal injection of LPS both caused the leakage of mtDNA into the cytoplasm in retinal tissue. After the transfection of mtDNA in vivo, the levels of cGAS, STING, and IFN-β mRNAs and the protein levels of STING, phosph-TBK1, phospho-IRF3, and IFN-β were upregulated. mtDNA injection also induced the activation of caspase 3 and caspase 9. BAX and BAK were increased at both the mRNA and protein levels. The release of cytochrome c from the mitochondria to the cytosol was increased after mtDNA injection. The wave amplitudes on ERG decreased and retinal cell apoptosis was detected after mtDNA injection.

**Conclusions:**

Cytosolic mtDNA triggers an inflammatory response. It also promotes apoptosis and the dysfunction of the retina.

**Supplementary Information:**

The online version contains supplementary material available at 10.1186/s40659-022-00390-6.

## Background

Mitochondria have increasingly been implicated as the gatekeepers of cell fate, with decisive roles in diverse cellular responses, including apoptosis, autophagy, and innate immunity [1, 2]. Under normal conditions, the mitochondrial DNA (mtDNA) is strictly packed in the mitochondrion [3]. However, pathological stimuli cause mitochondrial damage and mtDNA leakage [4]. The release of mtDNA has been reported in various pathological conditions, including rheumatoid arthritis [5], cardiovascular diseases [6], mechanical and hypoxic injury (passive mechanism) [7], and systemic inflammation [8]. In previous research, we found that lipopolysaccharide, hydrogen peroxide (H_2_O_2_) or high d-Glucose induced mtDNA escape into the cytosol of retinal microvascular endothelial cells (RMECs) [4]. On entering the cytoplasm, mtDNA acts as a damage associated molecular pattern (DAMP), cell-type- and context-specifically engaging multiple pattern recognition receptors to trigger proinflammatory and type I interferon (IFN) responses [9]. It has been reported that cytosolic mtDNA is involved in the activation of cyclic GMP–AMP synthase (cGAS)–stimulator of interferon response cGAMP interactor (STING)-driven IFN signaling [10]. In RMECs, cytoplasmic mtDNA is recognized by cGAS, increasing the expression of inflammatory cytokines through the STING–TBK1 signaling pathway. In age-related macular degeneration (AMD), the release of mtDNA from the retinal pigment epithelium (RPE) mitochondria activates cGAS and drives noncanonical inflammasome activation, resulting in RPE degeneration [11].

In the present study, we further explored the retinal damage caused by cytosolic mtDNA in vivo. To simulate the release of mtDNA in the cytoplasm of retinal cells under pathological conditions, we introduced the mtDNA extracted in vitro into cells using the plasmid transfection method, and further explored the mechanism of mtDNA-mediated cell damage and retinal functional impairment.

## Methods

### Animals and induction of retinal injury by light or lipopolysaccharide (LPS)

The animal protocols used in the study were approved by the Animal Ethics Committee of the Eye and Ear Nose Throat Hospital of Fudan University, Shanghai, China and the experiments complied with the Association for Research in Vision and Ophthalmology’s Statement on the Use of Animals in Research. Sprague–Dawley rats (n = 85, male, 6–8 weeks old, approximately 200 g) were kept in a colony room on a 12-h light/12-h dark cycle at 22–24 °C. Normal food and water were available ad libitum. All experiments were performed on the animals after an intraperitoneal injection of 10% chloral hydrate (0.4 ml/100 g). At the end of the experiments, the animals were anesthetized with an overdose of 10% chloral hydrate and killed by cervical dislocation. All operations were performed in such a way as to minimize animal suffering. Before light exposure, the animals’ pupils were dilated with 1% atropine eye drops (Santen Pharmaceuticals Co., Ltd, Osaka, Japan). In the light-injury model, the rats were separated into individual transparent boxes with wire tops and exposed continuously to bright light (5000 lx) in a light box to induce retinal degeneration. After exposure for 24 h, the rats were returned to the normal light/dark cycle and room conditions. In the intravitreal LPS injection model of retinal damage, phosphate-buffered saline (PBS; HyClone, Logan, UT, USA) was used to dissolve and dilute LPS (Sigma-Aldrich, St. Louis, MO, USA) to a concentration of 125 ng/µl. Rats were anesthetized and the pupils were dilated with atropine sulfate (Santen Pharmaceuticals Co., Ltd). Then, a Hamilton microinjector (Hamilton, Reno, NV, USA) was used to perform the intravitreal injection with LPS (2 µl, 125 ng/µl) at 1 mm posterior to the limbus. The right retinal samples were collected before exposure or 1, 3, 5, and 7 days after light- or LPS-induced injury for further analysis.

### Isolation of cytosolic and mitochondrial fractions and detection of cytosolic mtDNA

The anterior segment of each eye was removed and the retina was isolated after the eye had been enucleated after light- or LPS-induced injury, according to the experimental schedule. The retinas were weighed and cut up. Each fresh retina (usually ≤ 1 h after the animal was killed) was processed with the Mitochondria Isolation Kit for Tissue (C3606, Beyotime, Shanghai, China). One of our studies in the same period has experimented with the same method to extract mitochondria and verified the purity of mitochondria (Additional file 1: Fig. S1). In this study, we do not repost the results of purity of cytosolic and mitochondrial fractions. The tissue was then mixed with 10 times the volume of mitochondrial separating reagent A and homogenized with a Dounce tissue grinder (10 passes). The homogenates were centrifuged at 600×*g* for 10 min at 4 °C. The supernatants were pipetted, transferred to fresh 1.5 ml tubes, and centrifuged at 11,000×*g* for 10 min at 4 °C. The mitochondria precipitation was isolated from the cytosolic supernatant. The cytosolic supernatant without mitochondria was used for subsequent experiments according to the Mitochondria Isolation Kit for Tissue (C3606, Beyotime, Shanghai, China). All experimental procedures were performed on ice. The PCR assay was used to detect the cytosolic mtDNA, as described in our previous work [4]. In brief, the above-mentioned mtDNA was mixed with Buffer FG1 and Buffer FG2 of the FlexiGene DNA Kit (no. 51206, Qiagen) in a 2 ml tube. After inverting the tube three times, the mixture was incubated at 65 °C for 10 min. Isopropanol (100%) was used to induce DNA precipitation. The tube was centrifuged for 3 min at 10,000×*g*. Then, Buffer FG3 was used to dissolve the DNA precipitation at 65 °C for 30 min. Quantitative PCR (qPCR) was used to amplify the sequence encoding mitochondrial cytochrome c oxidase 1 (*mt-Co1*) to detect the mtDNA and to amplify the 18*S* rDNA sequence to detect the nuclear DNA. The levels of mtDNA were normalized to the nuclear DNA and compared between groups. The primer sequences are listed in Additional file 2: Table S1. According to previous studies, most methods for calculating the copy number of mtDNA was measured and normalized with the copy number of nuclear DNA. And these methods did not perform quantitative calculations, but rather calculate relative ratios. We selected the 18S rDNA sequence as the reference according to these methods [11–13].

### Preparation and retinal transfection of mtDNA

The mtDNA was isolated from normal rat retinal cells as previously described [4]. According to the Mitochondrial DNA Isolation Kit (ab65321, Abcam, Cambridge, MA, USA), the isolated mtDNA was circular mtDNA about 15–20 kDa. The rat retinal microvascular endothelial cells were collected, washed with ice-cold PBS, and resuspended in 1× Cytosol Extraction Buffer. After incubation on ice for 10 min, the cells were homogenized with an ice-cold Dounce tissue grinder. The homogenized mixture was centrifuged at 700×*g* for 10 min at 4 °C. The supernatant was decanted into a new 1.5 ml microcentrifuge tube and centrifuged at 10,000×*g* for 30 min at 4 °C. The pellet was resuspended in 1× Cytosol Extraction Buffer and centrifuged again at 10,000×*g* for 30 min at 4 °C, after which the supernatant was discarded. The pellet was resuspended in Enzyme Mix (5 µl) and incubated in a 50 °C for at least 60 min. The mixture was then centrifuged at 12,500×*g* for 5 min. The supernatant was discarded and the pellet (mtDNA) was resuspended in Tris–EDTA (TE) buffer. The mtDNA was then diluted to a concentration of 1 µg/µl for the subsequent experiments. A solution (0.02 µg/µl mtDNA) containing 1 µl of mtDNA (1 µg/µl), 47.5 µl of PBS, and 1.5 µl of Attractene Transfection Reagent (Qiagen, 301005) was prepared and incubated at room temperature for 15 min before intravitreal injection. The control solution contained 48.5 µl of PBS and 1.5 µl of Attractene Transfection Reagent (Qiagen, 301005). According to our previous study [4] in retinal microvascular endothelial cells stimulated with mtDNA, the concentration of mtDNA solution of intravitreal injection was 0.02 µg/µl. The rats were anesthetized and injected intravitreally with the mtDNA solution (0.02 µg/µl, 2 µl) or control solution (2 µl) 1 mm posterior to the limbus. After stimulation with mtDNA or control solution, the rats were maintained under their original feeding conditions. To comply with ethical requirements, each rat was tested in only one eye and the right eye was selected for uniformity. In the experiment full-field electroretinography (ERG) as a noninvasive technique, the left eye was measured as the control. In full-field electroretinography (ERG), electrical currents can be affected by the environment and each mouse has individual differences. As reported in a previous study [14, 15], the right eye of each rat was injected intravitreally with mtDNA and the left eye was injected with control solution. In other experiments involving light injury, WB, PCR, and TUNEL, different animals were used as the controls.

### ERG

At 1, 3, 5, and 7 days after the intravitreal injection of mtDNA or control solution, retinal function was evaluated with ERG, recorded as a scotopic electroretinogram (Espion Electrophysiology System; Diagnosys LLC, Lowell, MA, USA). The rats were dark-adapted for 2 h and anesthetized. After the pupil was dilated with atropine sulfate (Santen Pharmaceuticals Co., Ltd), oxybuprocaine (Santen Pharmaceutical Co., Ltd) and carbomer (Bausch & Lomb, Rochester, NY, USA) were applied topically for corneal anesthesia and hydration, respectively. Under illumination with dim red light, platinum ring electrodes were placed on the corneal surface. A subdermal grounding electrode was placed hypodermically on the tail and an identical electrode inserted into the rat’s nose as the reference electrode. After 10 min dark adaptation, ERG signal recording was commenced as previously described [16]. The amplitude of the a-waves was measured from baseline to the troughs of the a-wave. The b-wave amplitude was measured from the negative peak of the a-wave to the positive peak of the b-wave.

### TUNEL staining

TUNEL staining was performed on the retinal sections using the In Situ Cell Death Detection Kit, TMR red (cat. no. 12156792910, Roche, Germany). The eyes were prepared and removed the anterior segment of the eye 7 days after the intravitreal injection of mtDNA or control solution. 4% paraformaldehyde was used to fixed the eyecups, and the eyecups were then dehydrated in sucrose solutions (20% for 2 h and 30% for overnight). The tissues were embedded and stored at − 80 °C and sagittal sections cut (10 μm). The tissue sections were fixed with 4% paraformaldehyde for 20 min at room temperature. The sections were immersed and washed in PBS for 30 min. The sagittal sections were then incubated with Permeabilization Solution (0.1% Triton X-100) for 30 min. The Enzyme Solution (vial 1) was added to the Label Solution (vial 2) to obtain the TUNEL reaction mixture (vial 1:vial 2 = 1:9). The sagittal sections were immersed in the TUNEL components and equilibrated for 60 min at 37 °C in a humidified atmosphere in the dark. After the sections were washed twice in PBS for 10 min each, 4′,6-diamidino-2-phenylindole (DAPI; Beyotime, Shanghai, China) was used to counterstain the samples. The slides were observed and photographed under a fluorescence confocal microscope (Leica TCS SP8 WLL, Wetzlar, Hesse-Darmstadt, Germany). The approximate excitation/emission peaks of the TUNEL stain and DAPI were 364/454 and 540/580 nm, respectively.

### Western blotting

The retinas were collected at 1, 3, 5, and 7 days after the intravitreal injection of mtDNA or control solution. The retinas processed with the Mitochondria Isolation Kit for Tissue as previously described [4] and the proteins collected from the supernatant and sediment were used for the detection of cytochrome c. Western blotting was performed according to methods described in previous study [4]. The following primary antibodies were used: rabbit anti-BAX (#2772, Cell Signaling Technology), rabbit anti-BAK (#12105, Cell Signaling Technology), rabbit anti-caspase 9 (AF6348, Affinity Biosciences Ltd), rabbit anti-cleaved caspase 9 (AF5240, affbiotech), rabbit anti-caspase 3 (ab44976, Abcam), rabbit anti-cleaved caspase 3 (ab49822, Abcam), anti-β-actin (ab8227, Abcam), anti-cGAS (ab179785, Abcam), rabbit anti-STING (D1V5L) (#50,494, Cell Signaling Technology), rabbit anti-TBK1/NAK (D1B4) (#3504, Cell Signaling Technology), rabbit anti-phospho-TBK1/NAK (Ser172) (D52C2) XP^®^ (#5483, Cell Signaling Technology), rabbit anti-IRF3 (D83B9) (#4302, Cell Signaling Technology), rabbit anti-phospho-IRF3 (Ser396) (D6O1M) (#29047, Cell Signaling Technology), anti-interferon β (ab140211, Abcam), rabbit anti-cytochrome c (10993-1-AP, Proteintech), and rabbit anti-VDAC1 (ab154856; Abcam). Three eyes from each group were tested.

## Real-time PCR

The retinas were isolated 1, 3, 5, and 7 days after the intravitreal injection of mtDNA or control solution. The total RNA was isolated from each retina with TRIzol Reagent (Invitrogen, Carlsbad, CA, USA), according to the manufacturer’s instructions, and quantified with a NanoDrop ND-1000 spectrophotometer (Thermo Fisher Scientific). Complementary DNA was synthesized with the PrimeScript RT Reagent Kit (Takara, Ohtsu, Shiga, Japan). A LightCycler^®^ 480 II Real-Time PCR instrument (Roche, Basel, Switzerland) was used to perform all real-time PCRs as reported in a previously published study [4]. The primer sequences are shown in Additional file 2: Table S1. Three eyes from each group were analyzed.

### Statistical analysis

Data were analyzed using the SPSS software version 17.0 (SPSS, Inc., Chicago, IL, USA). The ERG results are the means of six rats were used per experiment. Differences between two groups were compared with the Mann–Whitney *U* test. The Kruskal–Wallis test was used to compare differences among three or more groups. Values of P < 0.05 were considered statistically significant. At least three independent experiments were conducted for the quantitative results.

## Results

### Increased cytosolic mtDNA release in retina after injury

**Fig. 1:**
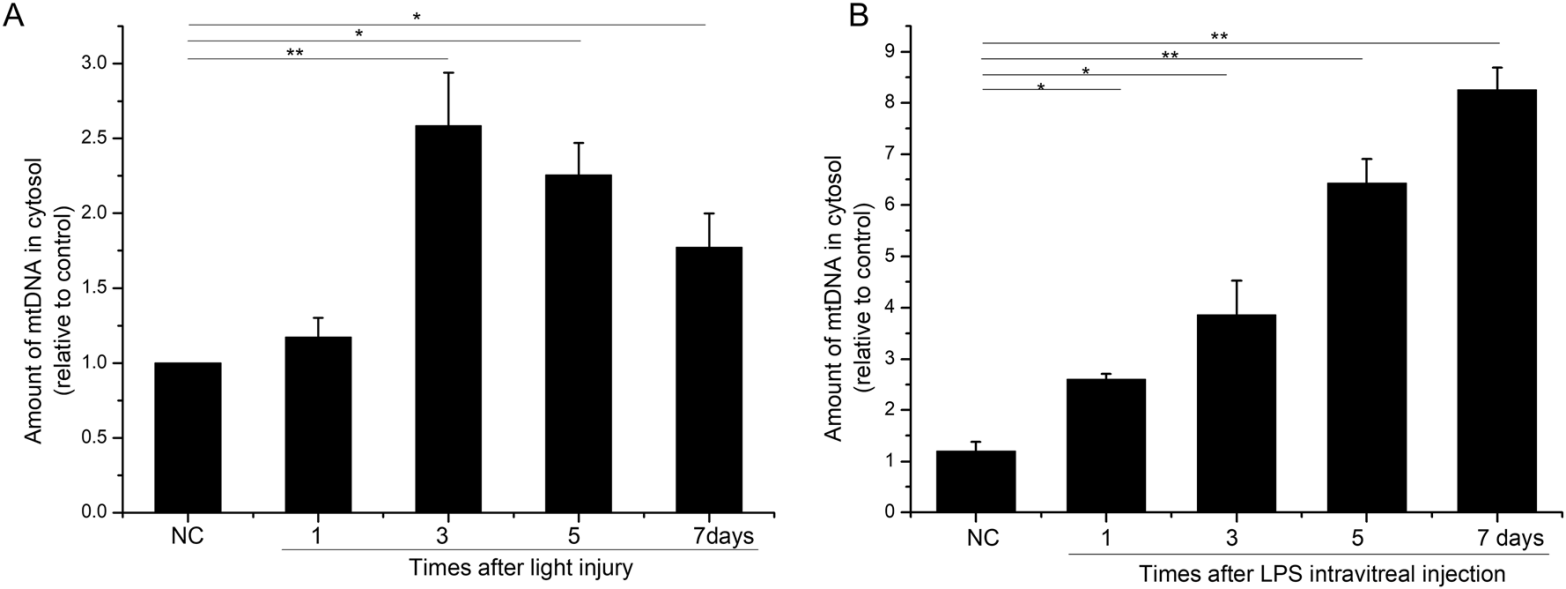
Real-time PCR was used to measure the copy numbers of mtDNA in the cytoplasm of retinal tissue. **A** Control and after light-exposure at 1, 3, 5, and 7 days; **B** control and an intravitreal injection of LPS at 1, 3, 5, and 7 days. *P < 0.05, **P < 0.01, n = 3 biological replicates in each group

The amount of cytosolic mtDNA in the retina, measured by PCR, was significantly increased at 3, 5, and 7 days after light injury (Fig. 1A). The released mtDNA in the retina was highest after 3 days, when it was 2.5 times higher than in the normal control (Fig. 1A). To provide further evidence that pathological stimuli cause the release of mtDNA, the cytosolic mtDNA was measured in rats after the intravitreal injection of LPS (125 ng/µl). As shown in Fig. 1B, the amount of cytosolic mtDNA in the retina increased after LPS injection and was maximal after 7 days compared with the control level (Fig. 1B).

**Fig. 2:**
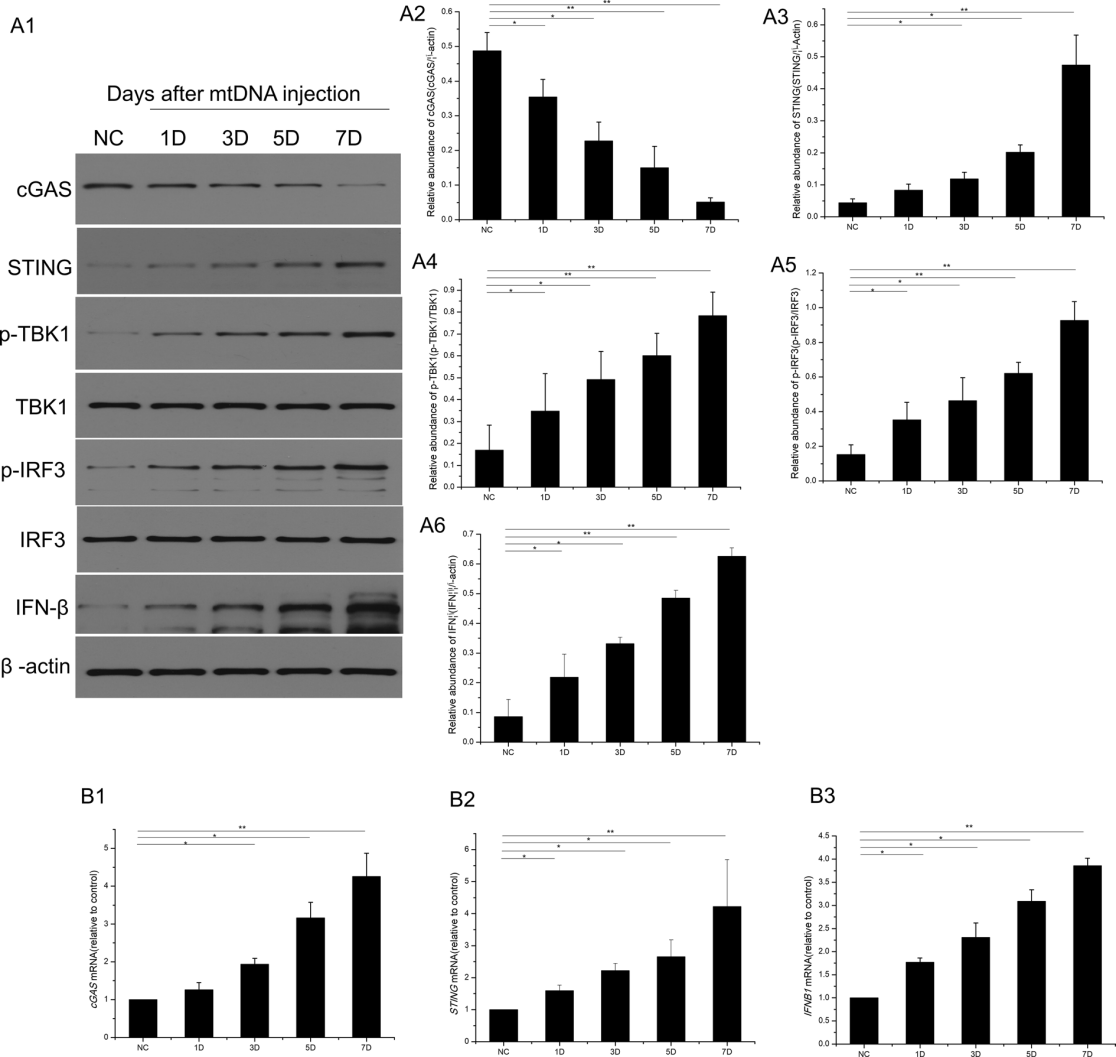
**A1** Western blotting was used to evaluate the changes in proteins in retinas at 1, 3, 5, and 7 days after mtDNA injection (0.02 µg/µl, 2 µl). **A2**–**A6** Western blotting analysis of cGAS, STING, phospho-TBK1, phospho-IRF3, and INF-β. **B1**–**B3** Real-time PCR was used to evaluate the transcription of cGAS, STING, and IFNB1. *P < 0.05, **P < 0.01, n = 3 biological replicates in each group

### The cGAS–STING–TBK1–IRF3 signaling pathway is activated by cytosolic mtDNA

After the retinal transfection of mtDNA, the transcription of cGAS mRNA increased significantly, as shown in Fig. 2B1. Cytosolic cGAS was then activated. The result is consistent with our previous findings [4] that in RMECs, cGAS decreased soon after mtDNA injection and cGAS mRNA increased after mtDNA injection. As a cytosolic DNA sensor, cGAS acts as a conserved enzyme and converts ATP and GTP into the dinucleotide cGAMP, which is a second messenger and activates the downstream cGAMP–STING signaling pathway. cGAS is then degraded by p62-depdendent ubiquitination, as previously reported [17]. Therefore, we hypothesized that the cGAS protein binds quickly to the mtDNA and is consumed after mtDNA injection. The transcription of *CGAS* then increases to increase subsequent protein translation. Therefore, consistent with previous research, the amount cGAS protein decreased gradually after mtDNA injection (Fig. 2A1, A2). STING, an endoplasmic reticulum (ER)-membrane protein, is activated by the cytosolic mtDNA–cGAS complex. After the retinal transfection of mtDNA, the transcription of *STING* increased (Fig. 2B2), accompanied by an increase in STING expression (Fig. 2A1, A3). The phosphorylation of TBK1 and IRF3 was significantly increased by stimulation with mtDNA at 1, 3, 5, and 7 days after mtDNA injection (Fig. 2A1, A4, and A5). Simultaneously, mtDNA injection also promoted the transcription and expression of IFN-β in the retina at 1, 3, 5, and 7 days after transfection (Fig. 2A1, A6, and B3).

### Retinal mtDNA innjection induced retinal apoptosis

**Fig. 3:**
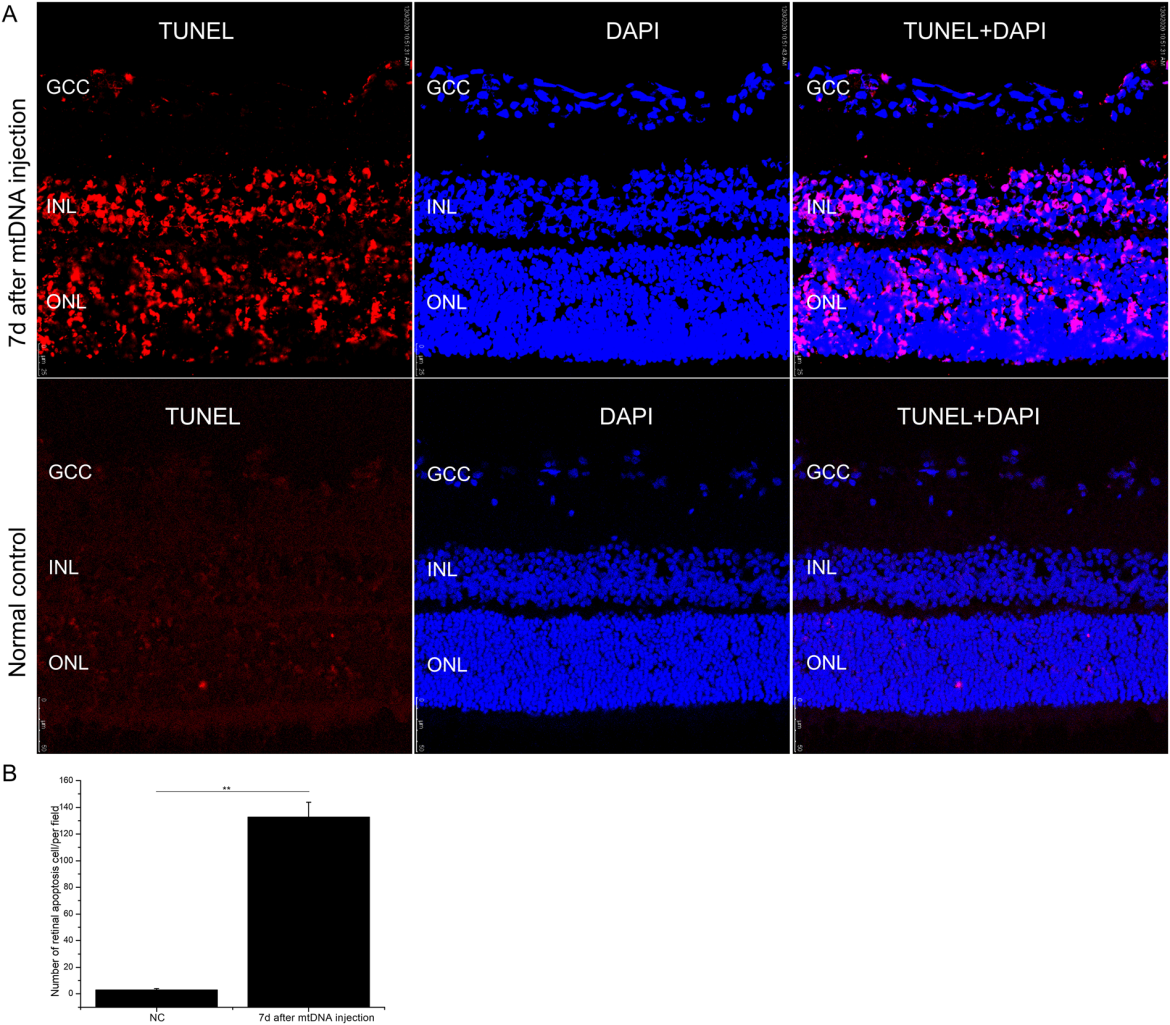
mtDNA injection (0.02 µg/µl, 2 µl) caused retinal cell damage, assayed with TUNEL staining after 7 days. **A** Representative retinal sections from the mtDNA retinal transfection group and normal control group. GCC, ganglion cell complex; INL, inner retinal nuclear layer; ONL, outer retinal nuclear layer. **B** Statistical analysis of the percentage of TUNEL-positive cells in each group. In each section, two areas 500 μm away from the optic nerve head, were selected for analysis. The number of TUNEL-positive cells was counted in each visual field and averaged. Only one section was chosen from each eye. n = 3 biological replicates in each group; **P < 0.01, scale bar: 50 μm

**Fig. 4:**
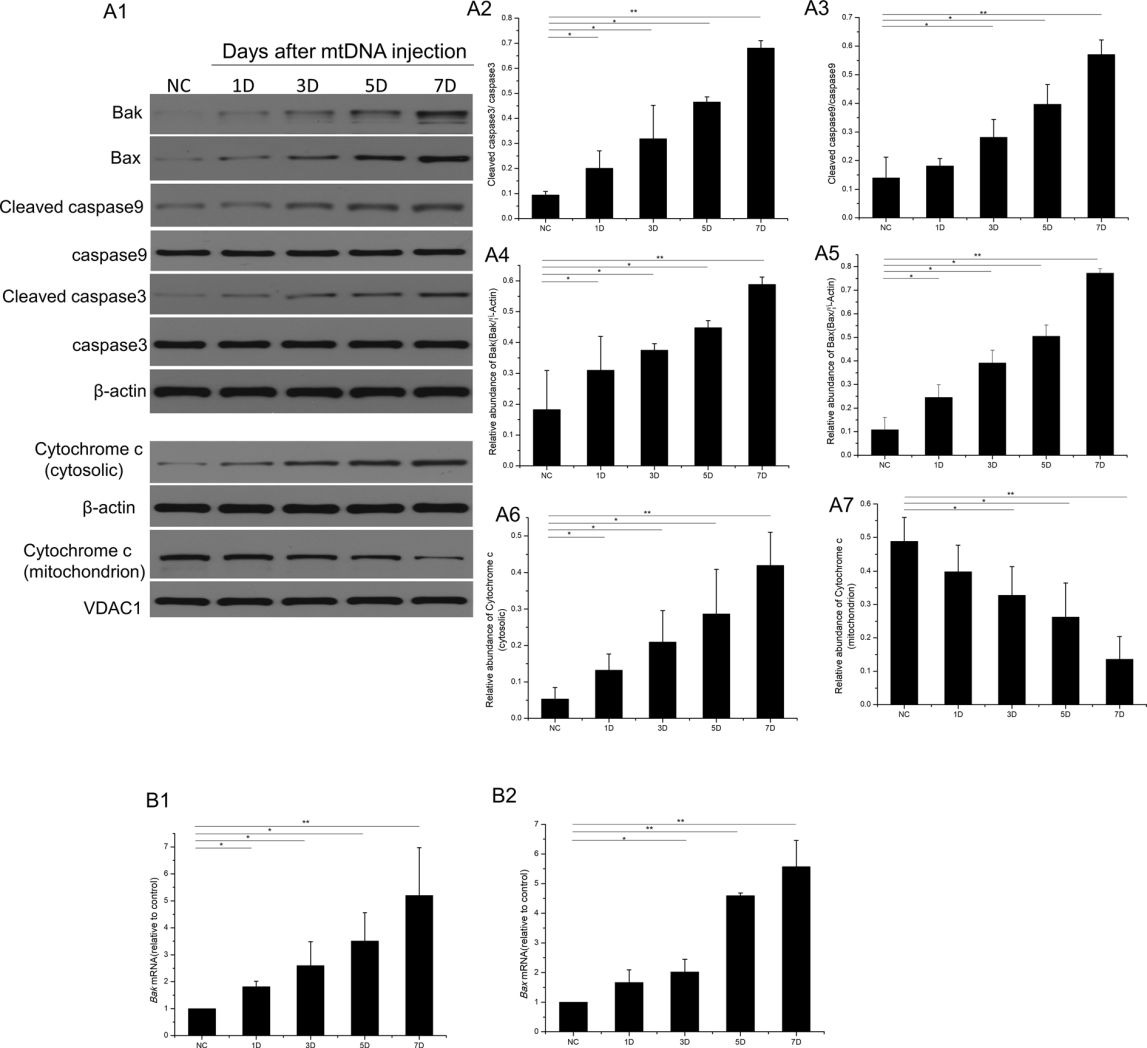
Mitochondrial apoptotic signaling pathway was activated by mtDNA injection (0.02 µg/µl, 2 µl). β-Actin was used as the loading control. VDAC1 was used as the internal control for mitochondrial proteins. **A1** Western blotting of retinal BAK, BAX, cleaved caspase 9, cleaved caspase 3, and cytochrome c protein levels in the normal control group and mtDNA-transfected groups after 1, 3, 5, and 7 days. **A2**–**A7** Western blotting analysis of cleaved caspase 3, cleaved caspase 9, BAK, BAX, cytochrome c in cytosolic and cytochrome c in mitochondrial. **B1**–**B2** Real-time PCR was used to evaluate the transcription of *Bax* and *Bak*. *P < 0.05, **P < 0.01, n = 3 biological replicates in each group

A TUNEL assay was used to assess the retinal cell damage at 7 days after the retinal transfection of mtDNA. As shown in Fig. 3, compared with the normal control, the number of TUNEL-positive nuclei was significantly increased in the transfected retinas, and they were located in the ganglion cell complex (GCC), inner retinal nuclear layer (INL), and outer retinal nuclear layer (ONL) (control group, 3 ± 1 TUNEL-positive cells; mtDNA-transfected group, 132.67 ± 11.24 TUNEL-positive cells; these are the total numbers of TUNEL-positive cells, P < 0.01). Consistent with the TUNEL staining results, the caspase 9/caspase3 signaling pathway was activated by mtDNA injection. Caspase 9 was activated and increased time-dependently on days 3, 5, and 7 after stimulation (Fig. 4A1–A3). Cleaved caspase 3 was also notably elevated 1 day after the intravitreal injection of mtDNA and peaked at 7 days. We then investigated the effects of caspase 9 and caspase 3 signal activation. The transcription and expression of proapoptotic proteins BCL-2-associated X (BAX) and BCL-2 antagonist/killer 1 (BAK) was significantly increased (Fig. 4A1,  A4–A5, B1–B2) at 1, 3, 5, and 7 days after retinal transfection with mtDNA, accompanied by the increasing release of cytochrome c from the mitochondria to the cytosol (Fig. 4A1, A6, A7).

### Retinal mtDNA injection causes retinal dysfunction

**Fig. 5:**
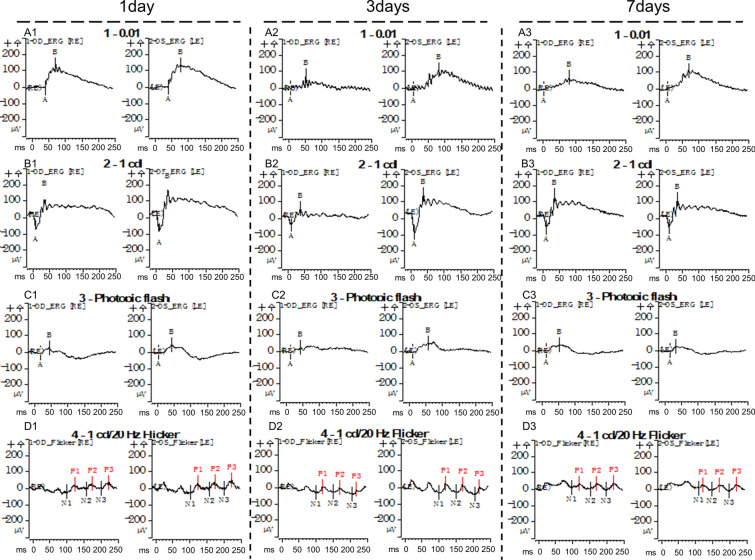
Retinal function at 1, 3, and 7 days after mtDNA injection. Representative images of a- and b-waves on rod-ERG, max-ERG, cone-ERG, and flicker-ERG in the control (left) eyes and mtDNA-treated (right) eyes were recorded, in that order, using the Espion Visual Electrophysiology system

**Fig. 6:**
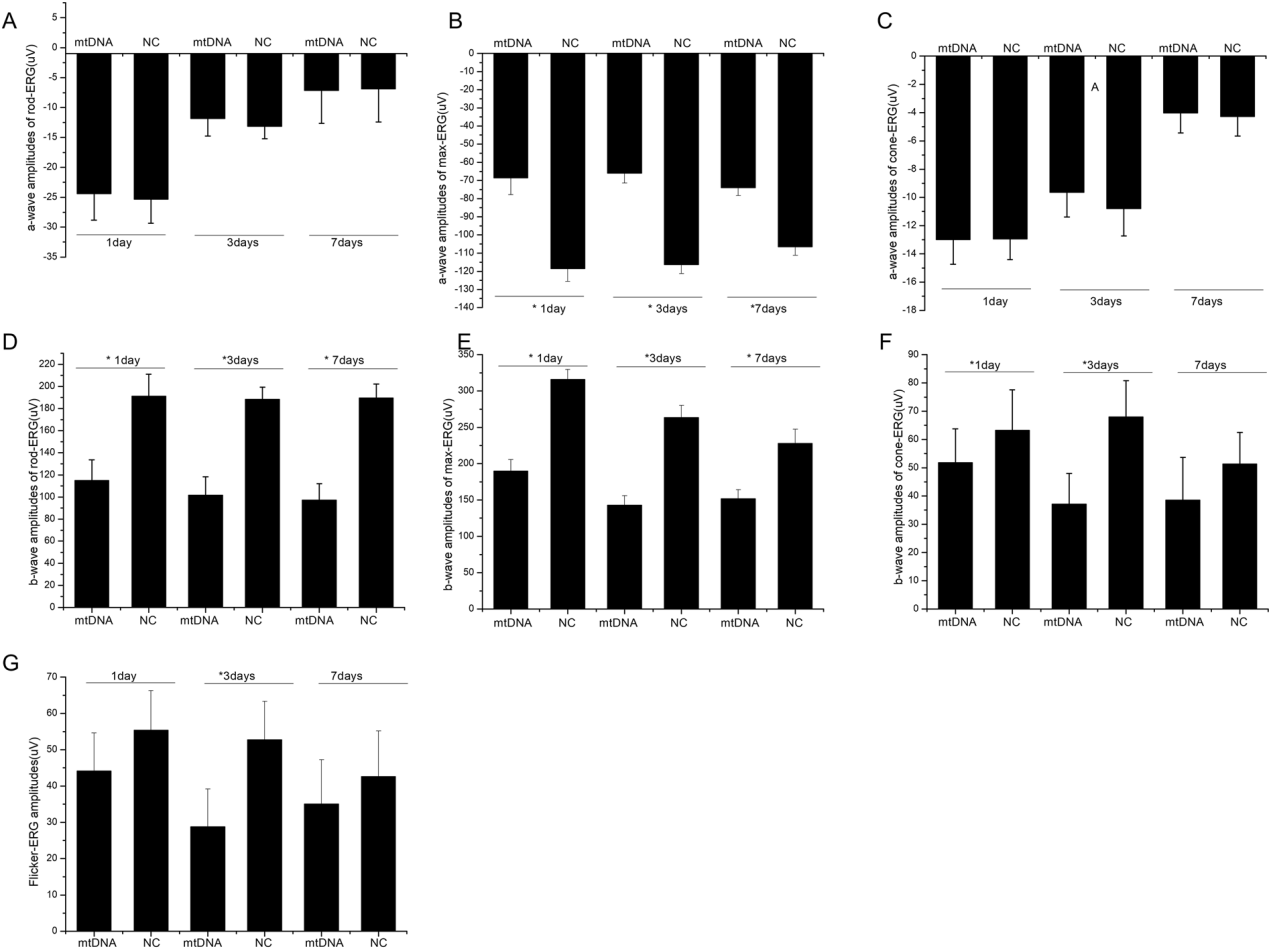
Statistical analysis of the amplitudes (µV) of the a- and b-waves on ERG at 1, 3 and 7 days after mtDNA injection (0.02 µg/µl, 2 µl). The right eye of each rat was intravitreally injected with mtDNA and the left eye of each rat was intravitreally injected control solution. **A** The a-wave amplitudes on rod-ERG. **B** The a-wave amplitudes on max-ERG. **C** The a-wave amplitudes on cone-ERG. **D** The b-wave amplitudes on rod-ERG. **E** The b-wave amplitudes on max-ERG. **F** The b-wave amplitudes on cone-ERG. **G** Flicker-ERG amplitudes. Data are means ± SD, n = 6 per group, *P < 0.05

ERG was used to evaluate retinal function after mtDNA injection. The representative images were recorded sequentially (Fig. 5). No significant differences were detected in the a-wave amplitude on rod-ERG of the mtDNA-transfected group and the normal control group (Fig. 6A). The amplitudes of the a-waves on max-ERG were significantly lower in the eyes after mtDNA injection than in the normal control eyes at 1 day (− 119.7 ± 45.1 vs. − 69.6 ± 40.2 µV, respectively; P < 0.05), 3 days (− 117.6 ± 43.5 vs. − 65.1 ± 53.2 µV, respectively; P < 0.05), and 7 days after mtDNA injection (− 101.2 ± 37.7 vs. − 76.1 ± 40.0 µV, respectively; P < 0.05) (Fig. 6B). Figure 6C shows that there were no significant differences in the a-wave amplitudes on cone-ERG between the mtDNA eyes and control eyes. The amplitudes of the b-waves on rod-ERG in the mtDNA-transfected rats were 128.1 ± 74.7 µV, 102.3 ± 97.3 µV, and 92.5 ± 48.0 µV on days 1, 3, and 7, respectively, whereas in the control eyes, the b-wave amplitudes were 185.7 ± 74.8 µV, 181.4 ± 99.4 µV, and 181.7 ± 91.3 µV, respectively (Fig. 6D) (all P < 0.05). Like the b-waves on rod-ERG, the amplitudes of the b-waves on max-ERG were significantly lower in the mtDNA-transfected eyes than in the control eyes, at 187.1 ± 91.6 µV, 131.1 ± 127.1 µV, and 143.5 ± 107.1 µV on 1, 3 and 7 days after the intravitreal injection of mtDNA, respectively (all P < 0.05; Fig. 6E). The b-wave amplitudes on cone-ERG (Fig. 6F) were also lower than in the control eyes on 1 day (52.3 ± 27.6 µV vs. 63.1 ± 20.3 µV, respectively; P < 0.05) and 3 days after mtDNA injection (36.1 ± 21.5 µV vs. 68.6 ± 28.5 µV, respectively; P < 0.05). Figure 6G shows that on flicker-ERG, the b-wave amplitudes were also reduced by mtDNA at 3 days relative to the control values (26.9 ± 16.2 µV vs. 54.5 ± 19.4 µV, respectively; P < 0.05). Therefore, mtDNA injection induces retinal dysfunction in rats.

## Discussion

Retinal degeneration occurs in a group of common retinal diseases that involve the progressive deterioration of retinal photoreceptor cells, eventually culminating in their death, which can lead to visual impairment [18]. The previous studies of our team found that light-injury and intravitreal LPS injection can lead to the inflammatory response or apoptosis in rats’ retina [15, 19, 20]. Exposure to bright light upregulated the activation of caspase-3 and caspase-9, which caused the apoptosis of photoreceptor cells and reduced the function of retina [16]. Despite recent advances in our understanding of the mechanisms underlying these retinal degenerative diseases, their molecular pathology remains unresolved, especially for age-related macular degeneration and retinitis pigmentosa [21, 22]. In the present study, we have demonstrated that mtDNA escapes from the mitochondria into the cytosol after retinal injury. We isolated this mtDNA and used it to transfect the retinas of rats. We found that cytosolic mtDNA is recognized by the DNA sensor protein cGAS, which triggers the activation of the STING–TBKI–IRF3–IFN-β signaling pathway. The increased secretion of IFN-β leads to the activation of inflammation and promotes retinal injury [23]. We also found that cytosolic mtDNA triggers the activation of the proapoptotic effector proteins BAX and BAK. After the activation of BAX and BAK, these proteins accumulate at the mitochondrial outer membrane and induce its permeabilization, causing the release of cytochrome c into the cytoplasm. This cytosolic cytochrome c then activates the formation of the apoptosome [24, 25]. Apoptosomes activate procaspase 9, which in turn activates the effector caspase 3, resulting in the activation of the apoptotic caspases [26, 27].

The cells of the retinal neuroepithelial layer are terminally differentiated neurons, with no regenerative ability. Noninfective inflammation plays an important role in retinal degeneration, such as the loss of retinal ganglion cells in glaucoma [28], the apoptosis of pigment epithelium cells in retinitis pigmentosa [29], the dysfunction of retinal photoreceptor cells in age-related macular degeneration [30], and even retinal detachment [31]. However, the exact mechanism of noninfectious inflammatory activation in the retina was still unclear. Recent studies have shown that cytosolic mtDNA can act as a DAMP and trigger proinflammatory and type I interferon (IFN) responses [32, 33]. After cellular stress or tissue injury, cells release mtDNA into the cytosol [33]. In our previous study, we found that pathological stimulation can induce mtDNA leakage into the cytosol of RMECs [4]. We also demonstrated that light injury and intravitreal LPS injection can lead to mtDNA release in the retina [4]. Cytosolic mtDNA is recognized by and binds to cGAS with intermolecular hydrogen bonds [34]. The binary cGAS–DNA complex then promotes the generation of cGAMP from GTP and ATP [35]. cGAMP also acts as a second messenger and activates the downstream cGAMP–STING signaling pathway. cGAS is degraded by p62-depdendent ubiquitination, as reported in previous research [17]. After the retina was transfected with mtDNA, the cGAS–STING–TBK1–IRF3 signaling pathway was activated and IFN-β accumulated. Therefore, we speculate that stimuli, such as light injury and intravitreally injected LPS, cause retinal cells to release mtDNA into their cytoplasm, which then activates the cGAS–STING signaling axis, mediating the inflammatory response and retinal damage.

Apoptosis is a noninflammatory form of cell death that has been identified in many diseases [36]. We have shown that retinal neuroepithelial cells, including retinal ganglion cells, bipolar cells, and photoreceptor cells, underwent apoptosis after the transfection of the retina with mtDNA, and that caspase 3 and caspase 9 were strongly activated. We also found that the transfection of mtDNA induced the transcription and translation of the proapoptotic molecules BAX and BAK, members of the BCL-2 family, which control and regulate the intrinsic (or mitochondrial) apoptotic pathway [37]. Once released into the cytosol, cytochrome c binds to apoptotic peptidase activating factor 1 (APAF1), activating the formation of the ring-like apoptosome [24, 25]. However, it is still unclear how cytosolic mtDNA promotes the upregulation of the proapoptotic molecules BAX and BAK. Researchers have shown that the BH3 domain of IRF3 interacts directly with BAX, and thus activates BAX through conformational changes [38–40]. Therefore, we infer that retinal transfection with mtDNA induces cell apoptosis through the cGAS–STING–TBK1–IRF3 axis [41]. How the complex regulation of apoptosis by mitochondrial damage and the release of mtDNA into the cytoplasm trigger apoptosis must be examined in detail in future research. About the results of TUNEL Staining, we suspect that cells in ONL and INL were more likely to be damaged by mtDNA stimulation, while GCC has a certain resistance to mtDMA stimulation. Or it takes longer for GCC cells to appear apoptosis, while the time we observed is shorter. This requires advancements to study the role of mtDNA in GCC cells. How the complex regulation of apoptosis by mitochondrial damage and the release of mtDNA into the cytoplasm trigger apoptosis must be examined in detail in future research.

These types of damage all lead to retinal dysfunction, either through the retinal inflammatory response or retinal neuronal apoptosis. We used ERG to evaluate the effects of cytosolic mtDNA on retinal function in vivo and detected significant attenuation of the wave amplitudes on ERG. Previous studies have pointed the ERG a-wave originates from photoreceptor cells, and the b-wave originates from bipolar cells [42, 43]. The flicker-ERGs might originate from the cone system, but it has not been confirmed [44–46]. Cone bipolar cells and rod bipolar cells are secondary neurons and mainly located in the inner retinal nuclear layer (INL). The lower amplitudes of the b-waves suggest possible damage to bipolar cells. According to the results of TUNEL, it can be observed that the INL undergoes apoptosis. The photoreceptor cell body is in the outer retinal nuclear layer (ONL) [47]. The weak changes of a wave may be related to the mild damage to the ONL. ERG was used to evaluate retinal function. We conclude that stimulation by mtDNA causes significant retinal dysfunctions in vivo.

In previous study, the release of mtDNA into the cytosol is due to the mitochondrial permeability transition pore (MPTP) opening which is triggered by matrix calcium. Cyclophilin-D (CyP-D) as a prominent mediator of the MPTP can regulate the opening of MPTP [48]. We hypothesize that MPTP opening could be prevented by interfering with CyP-D, thereby reducing mtDNA release into the cytoplasm and activation of the cGAS pathway. We cannot determine which cell type contributes the most to the release of mtDNA into the cytoplasm. In our previous researches, we found retinal microvascular endothelial cells can release mtDNA from mitochondria [4]. Another study found that RPE cells can also release mtDNA. This question is indeed a limitation of our experiment and we will continue to explore it in the future. We explored that mtDNA activates the cGAS-STNG pathway in previous in vitro cell experiments, so the main purpose of this study is to further verify this pathway in vivo. We did not explore other signaling pathways (such as AIM2, TLR9, ZBP1/DAI, NLRF3). And we will further study in future research. Some studies have found that cGAS is degraded by ubiquitination through P62 after activation [17]. We assumed that cytosolic cGAS was activated by the cytosolic mtDNA and was then degraded by P62-depdendent ubiquitination, as previously reported. The amount of cGAS protein in cells decreased but mRNA transcription increased. Finally, it is very likely TBK1-IRF3 can be also activated by other cytosolic DNA receptors resulting in the type 1 IFN response.

## Conclusions

Cytosolic mtDNA is recognized by the DNA sensor protein cGAS and triggers the cGAS–STING–TBKI–IRF3–IFN-β pathway, leading to the activation of the inflammatory response and the expression of the proapoptotic molecules BAX and BAK. This leads to the release of cytochrome c and the subsequent activation of caspases 3 and 9, causing mitochondrion-induced apoptosis and retinal dysfunction in vivo.

## Supplementary Information

**Additional file 1: Figure S1.** Western blotting of mitochondrion protein and cytosolic protein to verify the purity of mitochondria.

**Additional file 1: Table S1.** The primer sequences.

## Data Availability

Not applicable.
