# Supplementary material for: Intravitreal injection of mitochondrial DNA induces cell damage and retinal dysfunction in rats
Source: Biol Res. 2022 Jun 3;55:22. doi: 10.1186/s40659-022-00390-6 (PMC9164539; doi:10.1186/s40659-022-00390-6)
Supplement: Supplementary file 1 — Additional file 1: Figure S1. Western blotting of mitochondrion protein and cytosolic protein to verify the purity of mitochondria. [file 40659_2022_390_MOESM1_ESM.pptx]

## Slide 1
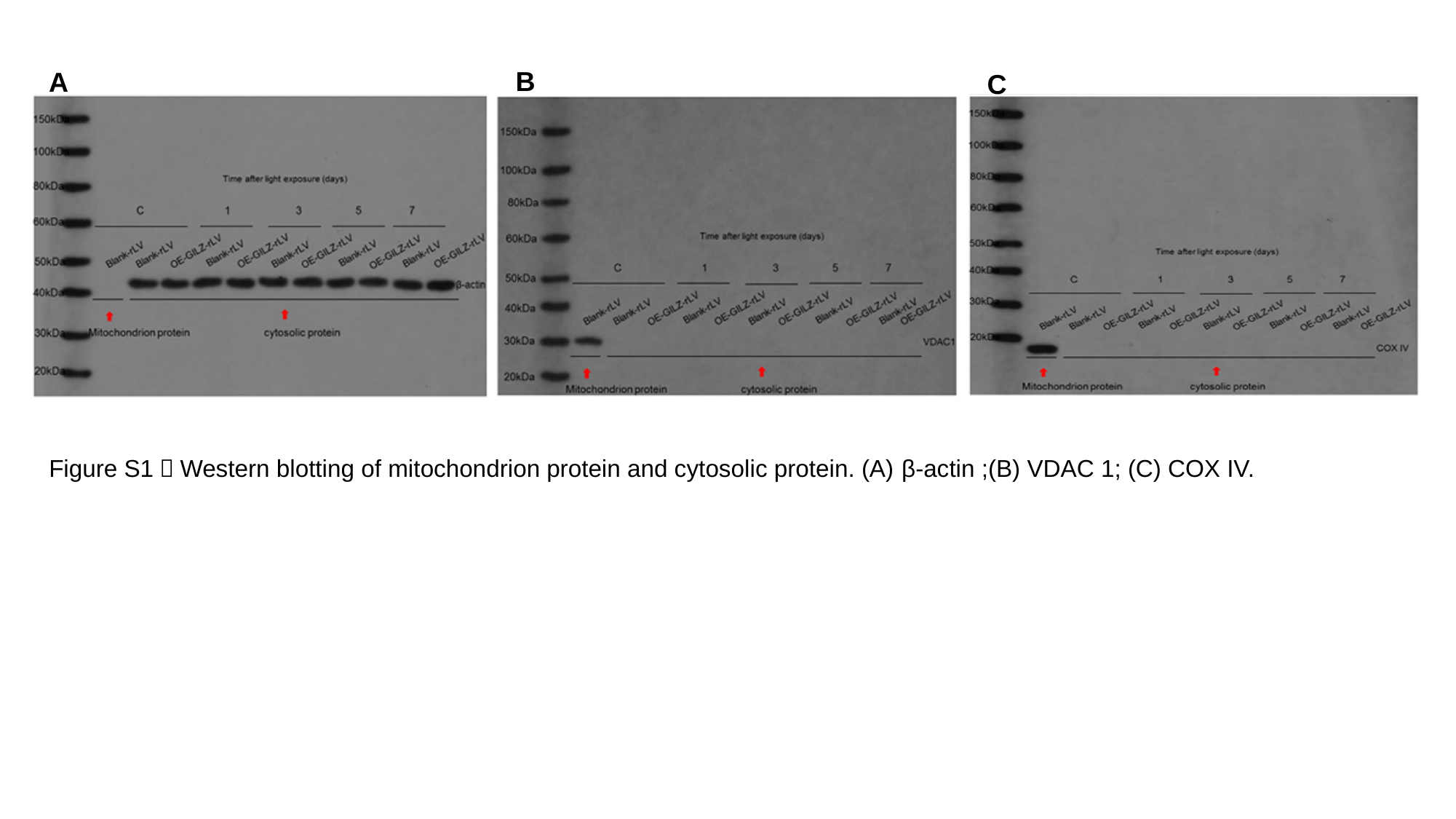

B
A
C
Figure S1：Western blotting of mitochondrion protein and cytosolic protein. (A) β-actin ;(B) VDAC 1; (C) COX IV.
