# Supplementary material for: Intravitreal injection of mitochondrial DNA induces cell damage and retinal dysfunction in rats
Source: Biol Res. 2022 Jun 3;55:22. doi: 10.1186/s40659-022-00390-6 (PMC9164539; doi:10.1186/s40659-022-00390-6)
Supplement: Supplementary file 2 — Additional file 1: Table S1. The primer sequences. [file 40659_2022_390_MOESM2_ESM.docx]

**Table S1**

| **Gene** | **Forward (5’→3’)** | **Reverse (5’→3’)** |
| --- | --- | --- |
| 18S rDNA | TAGAGGGACAAGTGGCGTTC | CGCTGAGCCAGTCAGTGT |
| mt-Co1 | GCCCCCGATATGGCGTTT | GTTCAACCTGTTCCTGCTCC |
| cGAS | GACTGGCTCGGCACAAAAGT | TAAGACGCCCACCTGTCTGG |
| STING | TGGGTCCTTGTGTGAGTCCTG | TGGATGCAGGTTGGAGTATGG |
| IFNB | CACTGGGTGGAATGAGACTATTG | TTGTCTTTAAGGTACCTTTGTACCC |
| Bax | TGAACTGGACAACAACATGGAG | AGCAAAGTAGAAAAGGGCAACC |
| Bak | CCAGCCTATTTAAGAGCGGC | CAAATTGGCCCAACAGAACC |
| actin | CGTTGACATCCGTAAAGACCTC | TAGGAGCCAGGGCAGTAATCT |
